# The ipsilesional DTI-ALPS index provides modest incremental prognostic value beyond clinical models for 90-day outcome in patients with acute MCA occlusion treated with intravenous thrombolysis: a prospective exploratory cohort study

**DOI:** 10.3389/fnagi.2026.1917297

**Published:** 2026-10-16

**Authors:** Lu Shen, Bo Ye, Jingye Lu, Chengying Yin, Huiqun Lu, Xiaojie Xu, Beijia Qin, Caixia Xu, Peng Yu

**Affiliations:** 1 Department of Ultrasound Diagnosis, Taixing People’s Hospital, Taixing, Jiangsu, China; 2 Department of Radiology, Taixing People’s Hospital, Taixing, Jiangsu, China; 3 Department of Neurology, Taixing People’s Hospital, Taixing, Jiangsu, China; 4 Department of Interventional Radiology, Taixing People’s Hospital, Taixing, Jiangsu, China; 5 Department of Psychology, Taixing People’s Hospital, Taixing, Jiangsu, China

**Keywords:** acute ischemic stroke, ALPS index, biomarker, magnetic resonance imaging, perivascular fluid dynamics, prognosis, thrombolysis

## Abstract

**Background and purpose:**

Impaired perivascular fluid dynamics may contribute to secondary brain injury after acute ischemic stroke, yet its prognostic relevance remains uncertain. We investigated whether the Diffusion Tensor Image Analysis along the Perivascular Space (ALPS) index—a quantitative MRI marker of perivascular fluid dynamics—provides incremental predictive value for long-term functional outcomes in patients with acute middle cerebral artery occlusion (MCAO) treated with intravenous thrombolysis.

**Methods:**

In this prospective observational cohort study, 128 patients underwent multimodal brain MRI within 24 h of admission, and the ALPS index was calculated in the hemisphere ipsilateral to the occlusion (ipsilesional ALPS). Favorable outcome at 90 days was defined as modified Rankin Scale (mRS) 0–2. Multivariable logistic regression identified independent predictors, with covariates (admission National Institutes of Health Stroke Scale [NIHSS] score, LDL cholesterol, and smoking status) prespecified on clinical grounds rather than selected by univariate screening. Incremental discriminative value was assessed by comparing nested models using receiver operating characteristic (ROC) analysis and the likelihood-ratio test (LRT), which is preferable to DeLong’s test for nested models. Calibration (Hosmer–Lemeshow test, Nagelkerke R^2^) and internal validation with 1,000 bootstrap resamples (optimism-corrected AUC) were performed.

**Interpretation:**

Lower admission NIHSS score [adjusted odds ratio (aOR) per 1-point increase: 0.890, 95% CI: 0.813–0.976; *p* = 0.013] and lower LDL cholesterol (aOR per 1 mmol/L: 0.660, 95% CI: 0.472–0.923; *p* = 0.015) were independently associated with favorable outcome, whereas the ipsilesional ALPS index showed a directionally consistent, non-significant association (aOR per 0.1-unit increase: 1.525, 95% CI: 0.948–2.454; *p* = 0.082). Adding the ALPS index to a base model containing NIHSS and LDL yielded a modest but significant improvement in discrimination (AUC 0.681 to 0.699; ΔAUC = 0.018; LRT *p* = 0.042); calibration was adequate (Hosmer–Lemeshow *p* = 0.31 and 0.28), and the gain persisted after bootstrap optimism correction (ΔAUC = 0.013).

**Conclusion:**

In this exploratory cohort, the ipsilesional ALPS index showed a consistent directional association with 90-day functional outcome and provided a modest but significant incremental improvement in discrimination beyond NIHSS and LDL cholesterol; the magnitude of this gain is small and requires external validation.

## Introduction

Acute ischemic stroke resulting from middle cerebral artery occlusion (MCAO) often leads to long-term disability, even when timely reperfusion is achieved via intravenous thrombolysis (IVT) (Binder et al., 2024). This clinical paradox suggests that factors beyond vascular recanalization, such as secondary neurotoxic cascades and impaired metabolic waste clearance (Jin et al., 2026), critically influence tissue recovery and functional outcomes.

The perivascular fluid dynamics, a paravascular network that facilitates cerebrospinal fluid influx and interstitial solute clearance, is a key regulator of post-ischemic brain homeostasis (Jamali et al., 2026). Preclinical studies demonstrate that acute cerebral ischemia disrupts aquaporin-4–dependent glymphatic flow, thereby promoting neurotoxic protein accumulation, exacerbating edema, and propagating injury to remote regions (Jiang-Xie et al., 2024; Jin et al., 2026). In human patients, cross-sectional studies have linked impaired perivascular fluid dynamics to greater stroke severity, motor impairment, and post-stroke cognitive decline (Perosa et al., 2022). Previous investigations, however, have been limited by heterogeneous stroke subtypes, assessments confined to subacute or chronic phases, or reliance on invasive imaging protocols unsuitable for routine clinical use.

The Diffusion Tensor Image Analysis along the Perivascular Space (DTI-ALPS) index offers a non-invasive, contrast-free method to quantify the directional diffusivity of water molecules along perivascular spaces relative to adjacent white matter as a surrogate for glymphatic activity using standard diffusion tensor imaging (DTI) (Li et al., 2024). The ALPS index was first established in Alzheimer’s disease (Taoka et al., 2017), where it detects early, stage-dependent alterations in perivascular fluid dynamics, supporting its utility as a transdiagnostic biomarker of perivascular brain clearance (Yu et al., 2025). In acute stroke, two recent human studies reported associations between reduced ALPS values and worse NIHSS scores or upper limb dysfunction (Kim et al., 2025; Lin, 2025). Three critical gaps remain: first, no study has focused exclusively on patients with hyperacute MCAO uniformly treated with IVT; second, the relationship between ipsilesional perivascular fluid dynamics and 90-day functional independence is uncharacterized; and third, the incremental discriminative value of the ALPS index beyond established predictors—such as admission NIHSS and lipid profile—has not been quantified in a prospective cohort.

It is well established that glymphatic system function naturally declines with advancing age. Therefore, in the context of acute ischemic stroke, distinguishing age-related baseline reductions in the ALPS index from stroke-induced acute glymphatic dysfunction is of critical importance. The present study aims to investigate whether the ipsilesional ALPS index, assessed during the acute phase (within 24 h of symptom onset), provides incremental prognostic value for 90-day functional outcome beyond conventional clinical indicators in patients with acute MCAO treated with intravenous thrombolysis. Age-related confounding is addressed separately through a dedicated age-stratified analysis.

To address these limitations, we conducted a single-center, prospective observational study of 128 patients with acute MCAO who received IVT within 4.5 h of symptom onset. Using the DTI-ALPS index derived from MRI performed within 24 h of admission, we aimed first to evaluate the association between the ipsilesional ALPS index and 90-day functional outcome, as measured by the modified Rankin Scale (mRS) (Figure 1). Our second aim was to quantify whether incorporation of this imaging biomarker provides measurable, albeit potentially modest, incremental discriminative value beyond conventional clinical models.

**Figure 1:**
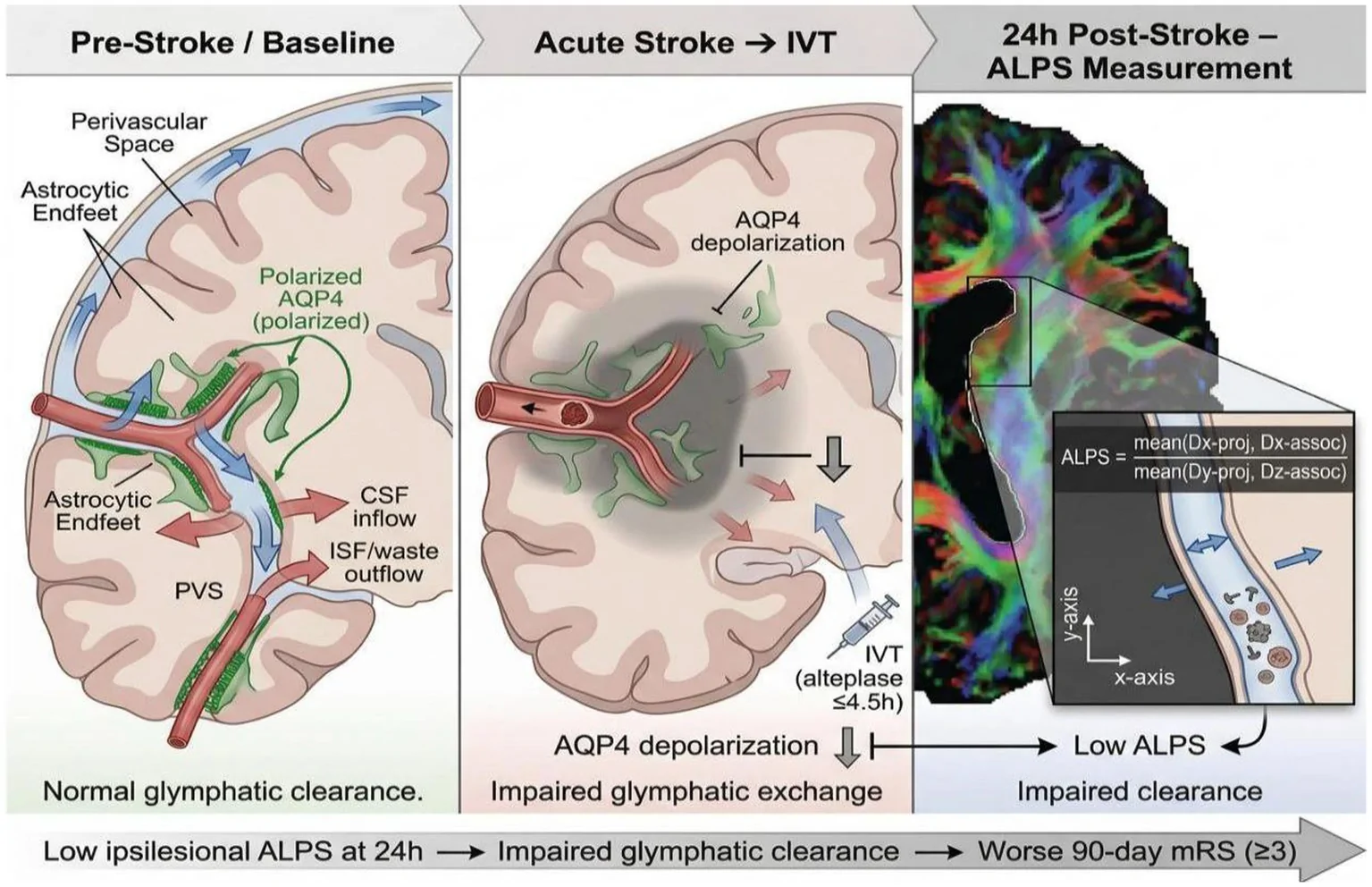
Proposed pathophysiological pathway linking DTI-ALPS index to long-term functional outcome after acute ischemic stroke.

## Materials and methods

### Study design and population

This single-center, prospective observational cohort study was conducted at the Stroke Center of Taixing People’s Hospital between February 2024 and December 2025. Consecutive patients diagnosed with acute ischemic stroke were screened for eligibility. Inclusion criteria were: (1) age ≥18 years; (2) confirmed occlusion of the middle cerebral artery (MCAO) on computed tomography angiography (CTA) or magnetic resonance angiography (MRA); (3) treatment with intravenous alteplase within 4.5 h of symptom onset (administered as a 0.9 mg/kg total dose, with 10% given as an intravenous bolus over 1 min and the remaining 90% infused over 60 min); and (4) completion of multimodal brain MRI, including diffusion tensor imaging (DTI), within 24 h of admission. Exclusion criteria comprised: non-MCA territory infarction; prior thrombectomy or endovascular treatment, or bridging endovascular thrombectomy after intravenous thrombolysis during the index hospitalization; contraindications to MRI; history of major intracranial disease (e.g., tumor, arteriovenous malformation); concurrent severe neurological disorders (e.g., Parkinson’s disease); incomplete clinical or imaging data; or loss to 90-day follow-up. The final analytical cohort consisted of 128 patients (Figure 2). The study protocol was approved by the Institutional Ethics Committee of Taixing People’s Hospital (Ethical Approval Number: LS2024055). The study was conducted in strict accordance with the Declaration of Helsinki (revised 2013). Written informed consent was prospectively obtained from all participants prior to MRI examination and data collection. For patients whose acute neurological deficits impaired their capacity to provide informed consent, written surrogate consent was obtained from their legally authorized representatives (next of kin) before enrollment. No waiver of individual informed consent was applied in this study.

**Figure 2:**
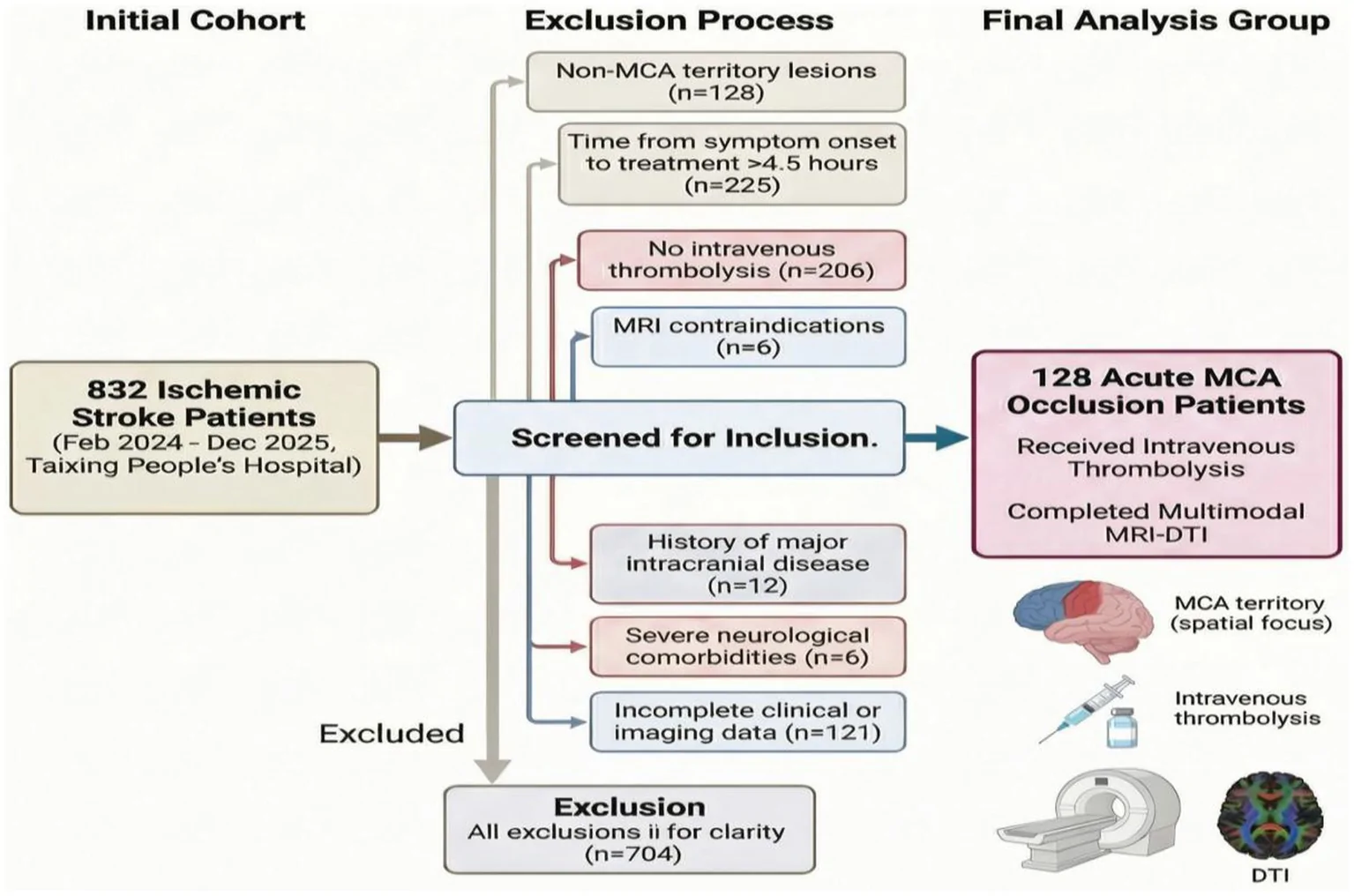
Flowchart of patient enrollment in the current study.

### Data collection and definitions

Baseline demographic and clinical characteristics were extracted from electronic medical records, including age, sex, smoking and alcohol consumption history, and comorbidities (hypertension, hyperlipidemia). Fasting blood samples were collected on admission for measurement of lipid profiles (LDL, HDL, triglycerides). Neurological severity was assessed using the National Institutes of Health Stroke Scale (NIHSS) by certified neurologists blinded to imaging biomarkers. The primary outcome was functional status at 90 days, evaluated via the modified Rankin Scale (mRS) through structured telephone interviews or outpatient visits by assessors blinded to baseline ALPS indices. For analytical purposes, a favorable outcome was defined as mRS 0–2 and coded as 1, while poor outcome (mRS 3–6) was coded as 0.

### Image acquisition and ALPS index calculation

All patients underwent brain MRI on a Siemens 3.0-T Vida scanner within 24 h (IQR: 6–24 h) after IVT administration. The diffusion tensor imaging (DTI) sequence was acquired using a single-shot echo-planar imaging (EPI) protocol with the following parameters: repetition time (TR) = 8,500 ms, echo time (TE) = 93 ms, slice thickness = 3 mm, field of view (FOV) = 220 × 220 mm, matrix = 128 × 128, b-value = 1,000 s/mm^2^, and 30 non-collinear diffusion-sensitizing gradient directions. Conventional T2-weighted images were also acquired (TR = 5,000 ms, TE = 98 ms). DTI data underwent standard preprocessing including eddy current correction and motion correction using FSL eddy. The ALPS index was calculated according to the method originally described by Taoka et al. (2017). As illustrated in Figure 3, regions of interest (ROIs) were placed in the lateral medullary white matter adjacent to the lateral ventricles on both hemispheres, avoiding perivascular spaces visible on T2-weighted images. Specifically, circular ROIs (diameter approximately 5 mm) were manually placed on the color-coded fractional anisotropy (FA) map at the level of the body of the lateral ventricles, where the projection fibers (corona radiata) run in the superior–inferior direction (blue color on the FA map) and the association fibers (superior longitudinal fasciculus) run in the anterior–posterior direction (green color). For each hemisphere, four ROIs were placed: two in the projection fiber area and two in the association fiber area, at identical slice levels on both sides to ensure symmetry. Care was taken to avoid regions with visible white matter hyperintensities, lacunar infarcts, or enlarged perivascular spaces on the co-registered T2-weighted images. Mean diffusivity values along the x-axis (Dxx), y-axis (Dyy), and z-axis (Dzz) were extracted for each ROI. The ALPS index was defined mathematically as: $\mathrm{DTI}-\text{ALPS index}=\frac{\text{mean}(\mathrm{Dxx}-\text{proj},\mathrm{Dxx}-\text{assoc})}{\text{mean}(\mathrm{Dyy}-\text{proj},\mathrm{Dzz}-\text{assoc})}.$ Here, Dxx-proj and Dxx-assoc denote the diffusion coefficients for projection and association fibers along the X-axis; Respectively, whereas Dyy-proj and Dzz-assoc correspond to the diffusion rates of projection fibers along the Y-axis and association fibers along the Z-axis.

**Figure 3:**
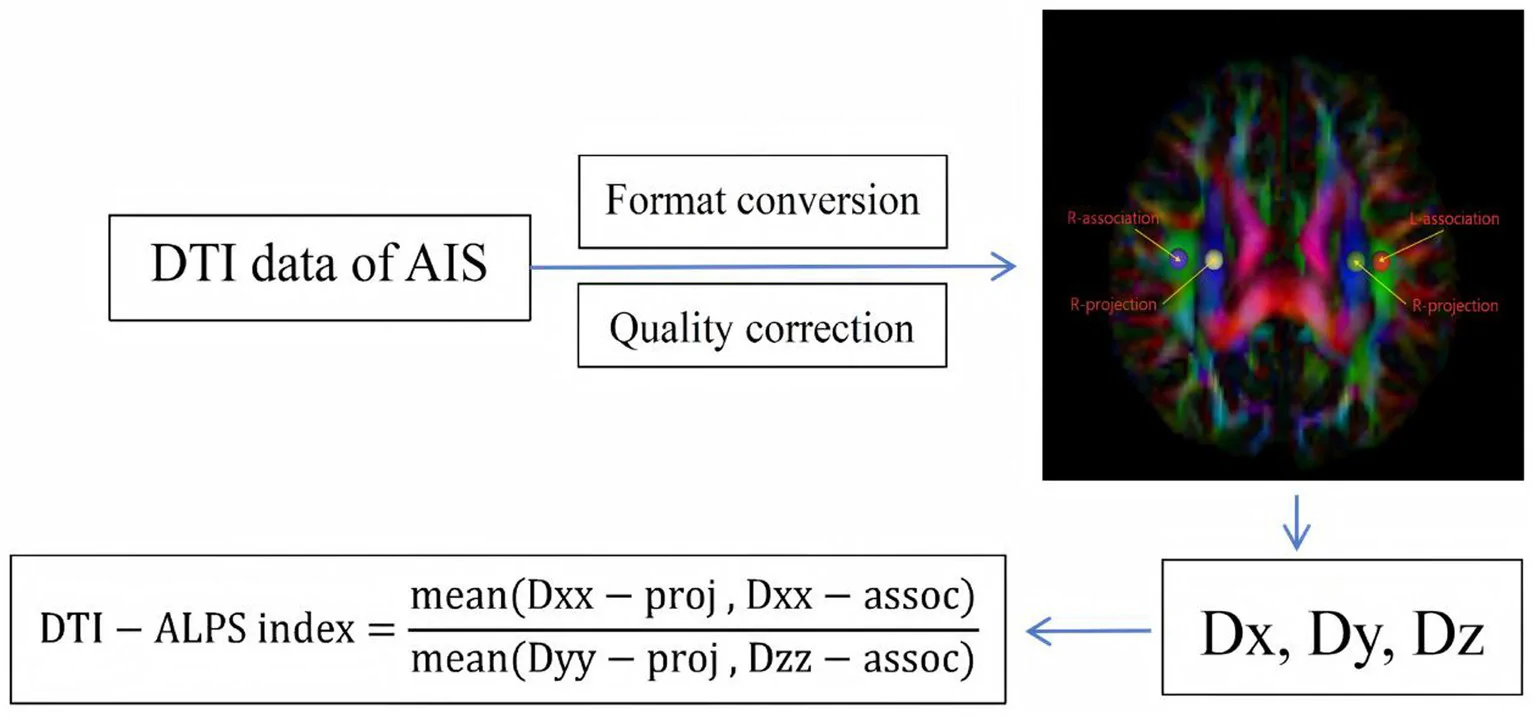
Flowcharts for ALPS index calculation.

The affected-hemisphere ALPS index (ipsilateral to the MCAO) was used as the primary imaging biomarker. All measurements were performed by two independent neuroradiologists blinded to clinical outcomes; inter-rater reliability was excellent (intraclass correlation coefficient = 0.92).

### Statistical analysis

Continuous variables were presented as mean ± standard deviation or median [interquartile range (IQR)] based on normality (Shapiro–Wilk test). Categorical variables were reported as frequencies (%). Group comparisons (favorable vs. poor outcome) were performed using Student’s *t*-test, Mann–Whitney U test, χ^2^ test, or Fisher’s exact test as appropriate.

To visualize the distribution of key continuous variables (affected-hemisphere ALPS index, admission NIHSS score, and LDL cholesterol) across outcome groups, we generated a double-Y-axis half-box plot with overlaid individual data points using Origin 2024 software (OriginLab, Northampton, MA, United States). This plot displays the interquartile range (box), median (central line), 5th percentile (star), outliers (crosses beyond 1.5 × IQR), and raw data points (jittered circles).

Multivariable logistic regression was employed to identify independent predictors of the 90-day functional outcome, modeled as favorable outcome (mRS 0–2). All covariates (admission NIHSS score, LDL cholesterol, and smoking status) were prespecified on the basis of established clinical relevance and biological plausibility, consistent with current guidance for prognostic-model studies; no data-driven univariate screening was used for variable selection. To facilitate clinical interpretability of the regression coefficients, the affected-hemisphere ALPS index was scaled per 0.1-unit increase; this rescaling changes only the unit of the coefficient and does not by itself address potential complete or quasi-complete separation. We therefore examined the data for separation by inspecting fitted probabilities and convergence diagnostics; no complete separation was identified, and as an additional sensitivity analysis, Firth’s penalized logistic regression was applied and yielded directionally consistent estimates for the ALPS index.

Model comparison was based on the likelihood-ratio test (LRT) for nested logistic-regression models, which is more appropriate than DeLong’s test for models fitted on the same data (Demler et al., 2012); DeLong’s test is reported only as a sensitivity analysis. Reclassification was summarized with the net reclassification improvement (NRI) using the category-free definition.

Model performance was evaluated by: (1) discrimination using the area under the receiver operating characteristic curve (AUC) with the likelihood-ratio test for nested-model comparison; (2) calibration via the Hosmer-Lemeshow goodness-of-fit test (χ^2^, *p* > 0.05 indicates adequate fit); and (3) explained variance (Nagelkerke R^2^). Internal validation was performed with 1,000 bootstrap resamples to compute the optimism-corrected AUC for each model. Four nested models were constructed to assess the incremental value of the ALPS index. Model A: NIHSS alone; Model B: NIHSS + LDL cholesterol; Model C: NIHSS + LDL + affected-hemisphere ALPS index; Model D: Full model including smoking status. All tests were two-tailed, and *p* < 0.05 was considered statistically significant. Analyses were performed using SPSS version 26.0 (IBM Corp.) and R version 4.3.0 (pROC package).

To evaluate the influence of aging on the ALPS index and its prognostic value, two exploratory analyses were performed. First, Pearson correlation analysis was used to assess the relationship between the ipsilesional ALPS index and age in the overall cohort. Second, patients were stratified by the age threshold of 70 years, which approximates the lower quartile of the cohort, into younger (< 70 years) and older (≥ 70 years) subgroups. The ipsilesional ALPS index was compared between favorable and poor outcome groups within each age stratum using the Mann–Whitney U test. Multivariable logistic regression was then performed within each stratum, adjusting for admission NIHSS score, LDL cholesterol, and smoking status (age served as the stratification variable and was therefore not entered as a covariate within each stratum).

## Results

### Patient baseline characteristics

A total of 128 patients with acute middle cerebral artery occlusion who underwent intravenous thrombolysis were included in the final analysis, stratified by their 90-day functional outcome as determined by the modified Rankin Scale (mRS): favorable outcome (mRS 0–2, *n* = 59) and poor outcome (mRS 3–6, n = 69). Baseline characteristics between the two groups are presented in Table 1. Patients with favorable outcomes were comparable to those with poor outcomes in terms of demographic features, including age (*p* = 0.933) and gender distribution (*p* = 0.38), as well as comorbidities such as hypertension (*p* = 0.73), alcohol use (*p* = 0.44), and hyperlipidemia (*p* = 0.90).

**Table 1:** Baseline characteristics of patients stratified by functional outcome at 90 days.

Variable	Favorable outcome (mRS 0–2, *n* = 59)	Poor outcome (mRS 3–6, *n* = 69)	*p-*value
Demographics			
Age (years)	74.0 [68.0–78.0]	75.0 [69.0–80.0]	0.933
Male, *n* (%)	34 (57.6)	45 (65.2)	0.379
Hypertension, *n* (%)	42 (71.2)	51 (73.9)	0.731
Smoking history, *n* (%)	21 (35.6)	30 (43.5)	0.370
Alcohol use, *n* (%)	12 (20.3)	18 (26.1)	0.444
Hyperlipidemia, *n* (%)	28 (47.5)	32 (46.4)	0.903
Clinical features			
Admission NIHSS score	12.0 [10.0–14.0]	14.0 [12.0–16.0]	**0.009**
LDL cholesterol (mmol/L)	3.02 [2.65–3.48]	3.21 [2.80–3.65]	**0.015**
HDL cholesterol (mmol/L)	1.25 [1.08–1.42]	1.18 [1.05–1.30]	0.122
Triglycerides (mmol/L)	1.78 [1.35–2.20]	1.65 [1.20–2.10]	0.188
NIHSS change (discharge − admission)	4.0 [2.0–6.0]	3.0 [1.0–5.0]	0.388
Imaging biomarkers			
ALPS index per 0.1 unit	8.1 [7.6–8.5]	7.8 [7.3–8.2]	**0.016**
Contralateral ALPS index	1.04 ± 0.03	1.02 ± 0.08	0.211

mRS, modified Rankin Scale; NIHSS, National Institutes of Health Stroke Scale; LDL, low-density lipoprotein; HDL, high-density lipoprotein; ALPS, Diffusion Tensor Image Analysis along the Perivascular Space. The ALPS index was scaled per 0.1-unit increment for clinical interpretability in regression models. Continuous variables with non-normal distribution are presented as median (interquartile range [IQR]); normally distributed variable (contralateral ALPS index) is presented as mean ± standard deviation. Categorical variables are presented as number (%). *p-*values were calculated using Mann–Whitney U test for continuous variables and chi-square test for categorical variables. Statistically significant results (*p* < 0.05) are in bold.

However, significant differences were observed in several clinical and imaging parameters. Notably, patients with poor outcomes had significantly higher admission NIHSS scores (*p* = 0.009), higher LDL cholesterol levels (*p* = 0.015), and impaired perivascular fluid dynamics as indicated by a lower affected-hemisphere ALPS index (*p* = 0.016). These baseline disparities are further illustrated in Figure 4, which visualizes the distribution of these three key continuous variables across outcome groups. The box-and-whisker plots reveal not only shifts in median values but also differences in data spread and the presence of outliers, highlighting the distinct profiles associated with favorable versus poor recovery.

**Figure 4:**
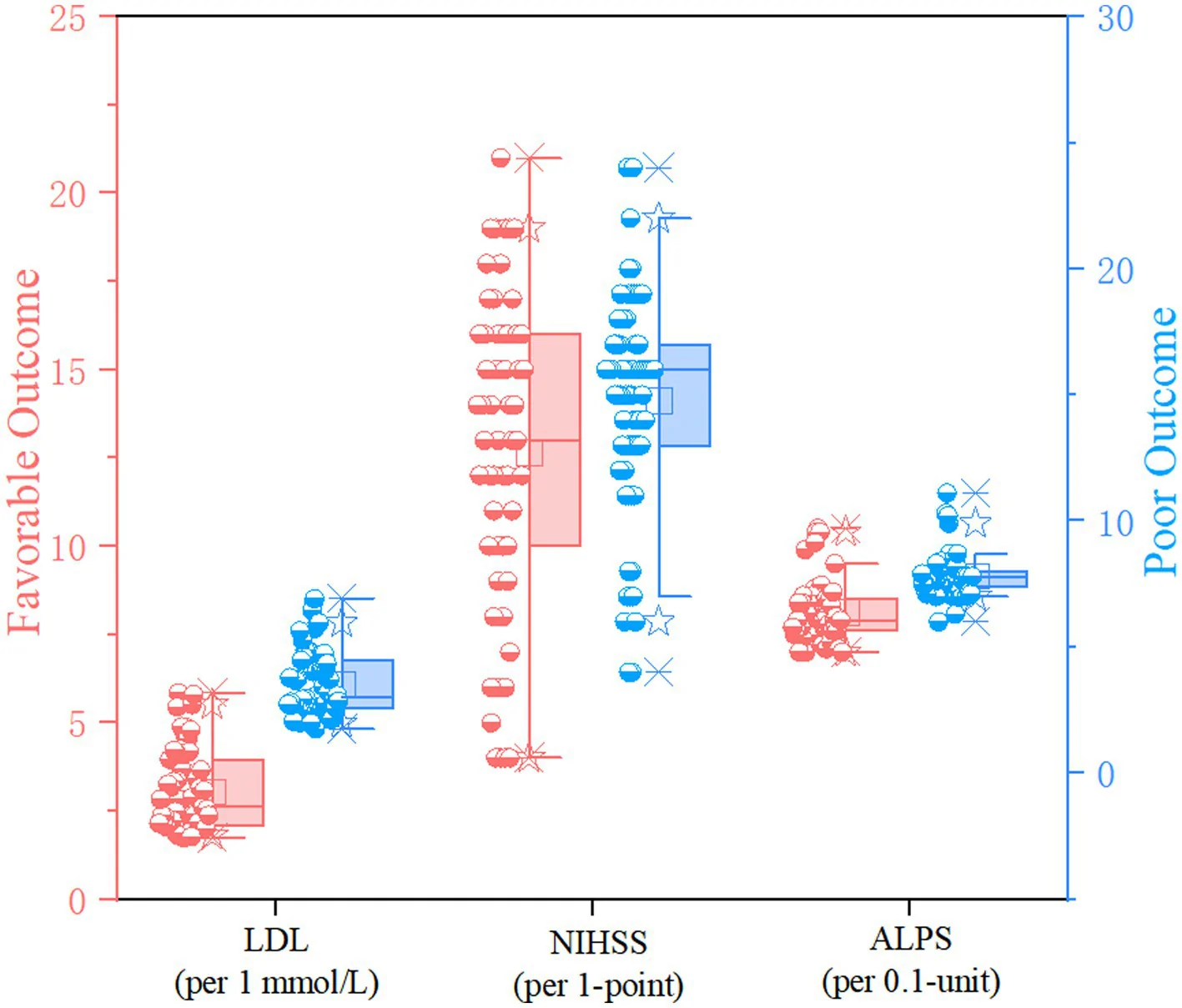
Distribution of key prognostic variables by 90-day functional outcome.

These findings provide the foundation for subsequent multivariable regression analyses aimed at identifying independent predictors of long-term functional outcome.

Note: Box plots represent the interquartile range (IQR), with the median indicated by the central line. Individual data points are shown as colored circles (red: favorable outcome, blue: poor outcome). Stars denote the 5th percentile values; crosses indicate outliers beyond 1.5 × IQR. The left y-axis corresponds to favorable outcomes (mRS 0–2), while the right y-axis corresponds to poor outcomes (mRS 3–6).

### Univariate analysis of potential predictors

We first evaluated the unadjusted association between each candidate variable and favorable functional outcome using univariate logistic regression (Table 2). Higher affected-hemisphere ALPS index (per 0.1-unit increment) was significantly associated with increased odds of favorable outcome (unadjusted OR = 1.602, 95% CI: 1.024–2.506, *p* = 0.039). Similarly, lower admission NIHSS score (per 1-point increase: OR = 0.897, 95% CI: 0.827–0.974, *p* = 0.010) was significantly associated with better 90-day outcome, and so was lower LDL cholesterol level (OR per 1 mmol/L increase = 0.718, 95% CI: 0.534–0.967, *p* = 0.029; equivalently, each 1 mmol/L increase in LDL cholesterol was associated with reduced odds of favorable outcome), consistent with the higher LDL levels observed in the poor-outcome group (Table 1). By contrast, current smoking (OR = 0.72, 95% CI: 0.36–1.45, *p* = 0.37) was not significantly associated with 90-day outcome in univariate analysis.

**Table 2:** Univariate logistic regression analysis for predictors of favorable functional outcome at 90 days.

Variable	Unadjusted OR (95% CI)	*p*-value
**Affected-hemisphere ALPS index (per 0.1-unit)**	**1.602 (1.024, 2.506)**	**0.039**
**Admission NIHSS score (per 1-point)**	**0.897 (0.827, 0.974)**	**0.010**
**LDL cholesterol (per 1 mmol/L)**	**0.718 (0.534, 0.967)**	**0.029**
Age (per 1-year)	0.989 (0.955, 1.025)	0.553
Smoking (yes vs. no)	0.72 (0.36, 1.45)	0.370
Hypertension (yes vs. no)	0.872 (0.400, 1.900)	0.731

OR, odds ratio; CI, confidence interval. The outcome variable was favorable functional outcome at 90 days defined as modified Rankin Scale (mRS) score of 0–2. All variables were analyzed using binary logistic regression. Statistically significant results (*p* < 0.05) are presented in bold.

### Multivariable analysis identifies independent predictors of outcome

To account for potential confounding, we constructed a multivariable logistic regression model with the prespecified covariates described in the Methods. After adjustment, two factors remained independently associated with 90-day functional outcome (Table 3): admission NIHSS score (adjusted OR = 0.890 per 1-point increase, 95% CI: 0.813–0.976, *p* = 0.013) and LDL cholesterol (adjusted OR = 0.660 per 1 mmol/L increase, 95% CI: 0.472–0.923, *p* = 0.015). The affected-hemisphere ALPS index demonstrated a positive but non-significant association (adjusted OR = 1.525, 95% CI: 0.948–2.454, *p* = 0.082), suggesting a potential neuroprotective role of perivascular fluid dynamics. In the three-covariate model used for the nested-model comparison (admission NIHSS score, LDL cholesterol, and the ALPS index), the ALPS estimate was aOR = 1.54 per 0.1-unit increase (95% CI: 1.01–2.35, *p* = 0.045), closely matching the likelihood-ratio test result (*p* = 0.042). Notably, inclusion of the ALPS index in the clinical model yielded a modest but statistically significant increase in AUC (ΔAUC = 0.018; likelihood-ratio test *p* = 0.042) and a significant category-free net reclassification improvement (NRI = 0.121, *p* = 0.036; Table 4). Hosmer–Lemeshow calibration was adequate for both the base and extended models (*p* = 0.31 and 0.28, respectively), and Nagelkerke R^2^ increased from 0.18 to 0.21 after adding ALPS. Bootstrap internal validation (1,000 resamples) yielded optimism-corrected AUCs of 0.65 and 0.66 for the base and extended models, respectively (ΔAUC = 0.013).

**Table 3:** Multivariable logistic regression analysis for clinical predictors and ALPS index in relation to 90-day functional outcome.

Variable	Adjusted OR (95% CI)	*P*-value
Affected-hemisphere ALPS index (per 0.1-unit)	1.525 (0.948, 2.454)	0.082
**LDL cholesterol (per 1 mmol/L)**	0.660 (0.472, 0.923)	**0.015**
**Admission NIHSS score (per 1-point)**	0.890 (0.813, 0.976)	**0.013**
Smoking (yes vs. no)	0.510 (0.231, 1.122)	0.094
ALPS index (3-variable model)*	1.54 (1.01, 2.35)	**0.045**

The multivariable model shown in Table 3 was fitted with favorable 90-day functional outcome (mRS 0–2) as the dependent variable, consistent with Tables 2, 4, and Figure 5. In this model, lower admission NIHSS score (aOR per 1-point increase = 0.890, 95% CI: 0.813–0.976, *p* = 0.013) and lower LDL cholesterol level (aOR per 1 mmol/L increase = 0.660, 95% CI: 0.472–0.923, *p* = 0.015) remained independently associated with favorable outcome; equivalently, higher NIHSS and higher LDL were associated with poor outcome. The ipsilesional ALPS index showed a directionally consistent but statistically non-significant association with favorable outcome (aOR per 0.1-unit increase = 1.525, 95% CI: 0.948–2.454, *p* = 0.082). *The estimate in the row “ALPS index (3-variable model)” comes from the three-covariate model (admission NIHSS score, LDL cholesterol, and the ALPS index) that was used for the nested-model likelihood-ratio test; its Wald *p-*value (0.045) closely matches the LRT *p-*value (0.042). Statistically significant results (*p* < 0.05) are presented in bold.

**Table 4:** Net reclassification improvement (NRI).

Category	Component	Estimate	95% CI	*p*-value
Event NRI (Patients with poor outcome, mRS 3–6)	Proportion correctly reclassified upward in risk	0.087	[0.012, 0.162]	**0.023**
Non-event NRI (Patients with favorable outcome, mRS 0–2)	Proportion correctly reclassified downward in risk	0.034	[−0.008, 0.076]	0.112
Total NRI	Event NRI + Non-event NRI	**0.121**	**[0.018, 0.224]**	**0.036**
ΔAUC	Change in AUC	**0.018**	**[0.001, 0.035]**	**0.042**

The reference model included admission NIHSS score and LDL cholesterol. The extended model further incorporated the ipsilesional ALPS index. The category-free NRI is the sum of the net reclassification proportions among events and non-events and therefore represents a composite of two proportions rather than a single proportion of patients reclassified; a category-free threshold of 0.5 was used for the base model as a sensitivity check. ΔAUC was tested with the likelihood-ratio test for nested models (DeLong’s test is reported as a sensitivity analysis). Calibration was assessed with the Hosmer–Lemeshow test (base model *p* = 0.31; extended model *p* = 0.28), and internal validation used 1,000 bootstrap resamples (optimism-corrected AUC: 0.65 vs. 0.66). The Event NRI is the proportion of patients with poor outcome (mRS 3–6) correctly reclassified upward in risk, and the Non-event NRI is the proportion of patients with favorable outcome (mRS 0–2) correctly reclassified downward in risk. Statistically significant results (*p* < 0.05) are presented in bold.

### Age-related analysis of the ALPS index

To investigate the influence of aging on the ipsilesional ALPS index and its prognostic value, we first examined the correlation between the ALPS index and age in the overall cohort. The ipsilesional ALPS index was significantly and negatively correlated with age (Pearson *r* = −0.31, *p* < 0.001), confirming that perivascular fluid dynamics decline with advancing age, consistent with prior reports in healthy and neurodegenerative populations(Fang et al., 2026). To distinguish age-related baseline decline from stroke-induced acute glymphatic dysfunction, we stratified the cohort into younger (< 70 years, *n* = 35) and older (≥ 70 years, *n* = 93) subgroups using a threshold approximating the lower quartile of the cohort’s age distribution. As shown in Table 5, the ipsilesional ALPS index was lower in the older subgroup [median 7.6 (7.1–8.0) in patients with poor outcome vs. 7.9 (7.4–8.3) in those with favorable outcome; *p* = 0.027] compared with the younger subgroup [8.1 (7.6–8.5) vs. 8.4 (7.9–8.8); *p* = 0.093], reflecting the expected age-related reduction in perivascular clearance. Importantly, within each age stratum, patients with favorable 90-day outcomes consistently exhibited higher ALPS indices than those with poor outcomes, and this directional difference was preserved across both subgroups.

**Table 5:** Ipsilesional ALPS index stratified by age group and 90-day functional outcome.

Subgroup	Favorable (mRS 0–2) N	ALPS Index (per 0.1 unit)	Poor (mRS 3–6) N	ALPS Index (per 0.1 unit)	*P*-value
Overall cohort (*N* = 128)	59	8.1 [7.6–8.5]	69	7.8 [7.3–8.2]	**0.016**
<70 years (*n* = 35)	17	8.4 [7.9–8.8]	18	8.1 [7.6–8.5]	0.093
≥70 years (*n* = 93)	42	7.9 [7.4–8.3]	51	7.6 [7.1–8.0]	**0.027**

The ipsilesional ALPS index is presented as median (IQR), scaled per 0.1-unit. Patients were stratified at the 70-year threshold, which approximates the lower quartile of the cohort’s age distribution. *p*-values were calculated using the Mann–Whitney U test to compare ALPS indices between favorable and poor outcome groups within each age stratum. In the overall cohort, the ipsilesional ALPS index was significantly and negatively correlated with age (Pearson *r* = −0.31, *p* < 0.001). Statistically significant results (*p* < 0.05) are presented in bold.

### Discriminative performance and incremental value of the ALPS index

To evaluate the clinical utility of these predictors, we assessed the discriminative performance of four nested prediction models using receiver operating characteristic (ROC) analysis (Figure 5). Nested-model comparisons were performed with the likelihood-ratio test, and calibration was assessed with the Hosmer–Lemeshow test. The baseline model including admission NIHSS alone showed modest discrimination (AUC = 0.634). Adding LDL cholesterol improved performance (AUC = 0.681), and further incorporation of the affected-hemisphere ALPS index yielded an incremental increase in AUC to 0.699. The full model, which also included smoking status, achieved the highest discriminative ability (AUC = 0.722). These results indicate that the ALPS index provides measurable incremental prognostic value beyond established clinical and laboratory markers in patients with acute ischemic stroke treated with intravenous thrombolysis.

**Figure 5:**
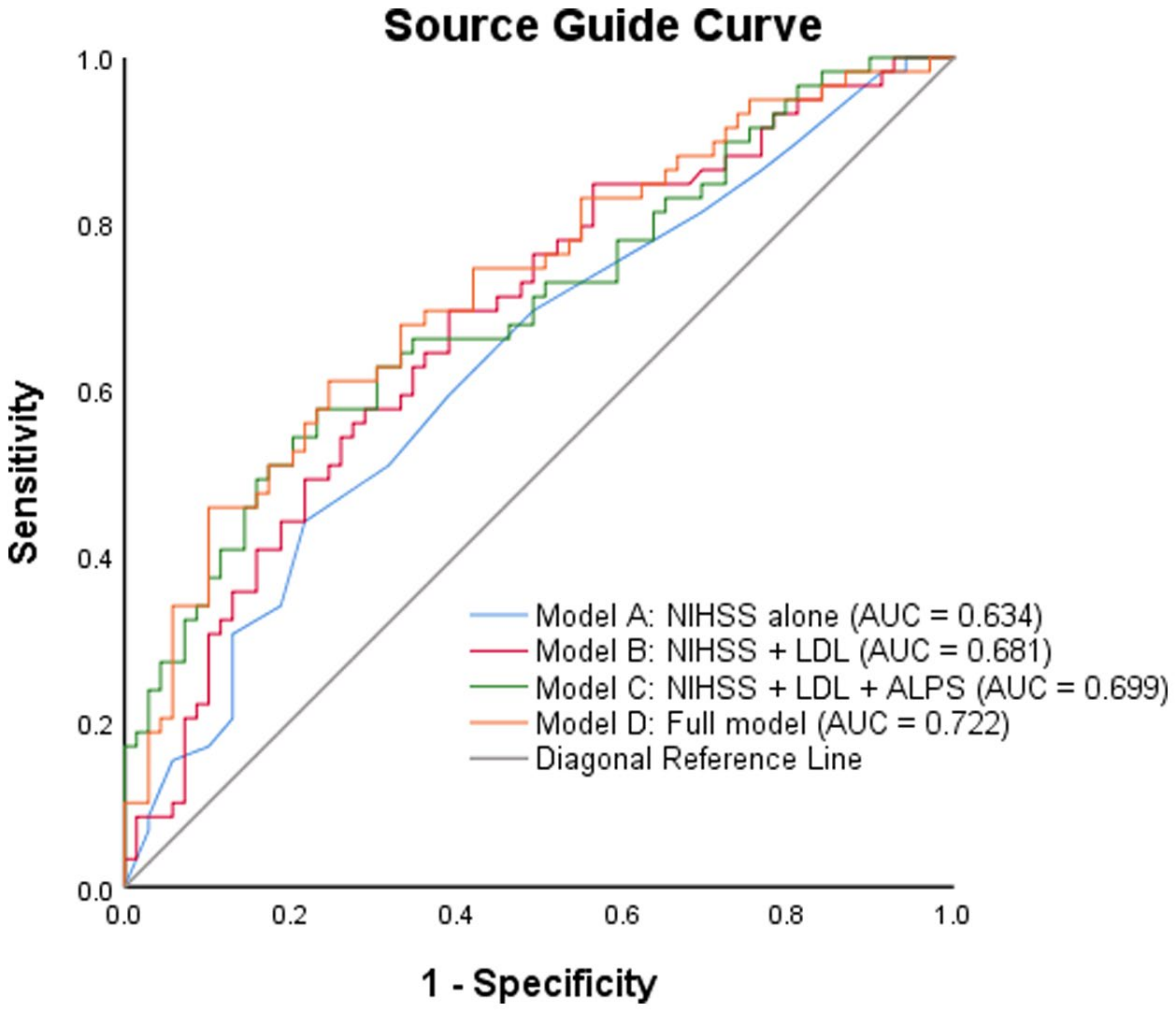
Receiver operating characteristic (ROC) curves for predicting favorable functional outcome at 90 days using four nested predictive models.

Receiver operating characteristic (ROC) curves comparing the discriminative performance of four sequential prediction models. Model A: NIHSS alone (AUC = 0.634), Model B: NIHSS + LDL cholesterol (AUC = 0.681), Model C: NIHSS + LDL + affected-hemisphere ALPS index (AUC = 0.699), Model D: Full model including smoking status (AUC = 0.722).

## Discussion

Acute ischemic stroke resulting from middle cerebral artery occlusion (MCAO) represents a leading cause of global adult disability and mortality, imposing substantial socioeconomic burdens (Lv et al., 2025). Although intravenous thrombolysis (IVT) within the 4.5-h window is the standard reperfusion strategy aimed at salvaging the ischemic penumbra, clinical outcomes remain highly variable even after successful recanalization (Ikenouchi et al., 2024; Konduri et al., 2021). This variability suggests that factors beyond mere vascular patency—such as secondary injury cascades involving impaired clearance of neurotoxic metabolites—critically influence tissue repair and long-term functional recovery. Consequently, identifying novel biomarkers capable of capturing these underlying pathophysiological mechanisms is essential for refining risk stratification and optimizing individualized management strategies in the acute setting.

Addressing this critical gap, our prospective observational cohort study investigated the prognostic utility of the ipsilesional Diffusion Tensor Image Analysis Along the Perivascular Space (DTI-ALPS) index, a non-invasive MRI metric reflecting perivascular fluid dynamics, in patients with acute MCAO treated with IVT. By evaluating perivascular fluid dynamics status within 24 h of symptom onset, we aimed to determine whether early system dysfunction predicts 90-day functional independence beyond conventional clinical predictors. Our findings reveal that while baseline stroke severity and lipid profiles remain dominant independent predictors, the incorporation of the ipsilesional ALPS index into established models yields a statistically significant improvement in discriminative performance. Although the absolute AUC improvement was modest (ΔAUC = 0.018; likelihood-ratio test *p* = 0.042), it translated into a statistically significant category-free net reclassification improvement (NRI = 0.121, *p* = 0.036), suggesting potential utility in refining individual risk estimates. The NRI is the sum of the net reclassification proportions among events and non-events, and therefore does not indicate that 12% of patients were correctly reclassified; rather, it reflects a composite net gain across the two outcome groups combined. These results underscore the potential of acute perivascular fluid dynamics assessment to provide incremental prognostic information, thereby highlighting its relevance as an emerging imaging biomarker in stroke outcome prediction.

Building upon the established role of baseline stroke severity, our finding that lower admission NIHSS scores independently predict favorable 90-day outcomes aligns with extensive literature confirming initial neurological deficit as a paramount determinant of tissue viability and recovery potential (Peng, 2025). However, the specific context of MCAO treated with IVT introduces unique pathophysiological constraints compared to broader acute ischemic stroke cohorts. While previous studies have identified various clinical and laboratory predictors of outcome after reperfusion therapy, including inflammatory markers such as the eosinophil-to-neutrophil ratio (Cai et al., 2021) and renal function (Park et al., 2022), our data reinforce that clinical severity remains the most robust anchor for prognostic modeling. The mechanism underlying this association likely involves the extent of irreversible core infarction at presentation, which dictates the ceiling for functional recovery regardless of successful recanalization. Furthermore, the consistency of NIHSS as a predictor across different vascular territories suggests that the volume of jeopardized parenchyma, rather than mere location, drives the secondary injury cascade that limits long-term independence (Fan et al., 2024).

In our cohort, higher admission LDL cholesterol was independently associated with a lower probability of favorable 90-day outcome, a direction of effect that is consistent with the conventional view of LDL as an atherogenic risk factor and with the higher LDL levels observed in the poor-outcome group (Table 1). This direction differs from reports of an apparent “cholesterol paradox” after stroke, in which low LDL levels have been linked to poor outcomes; however, that pattern is widely attributed to reverse causation and confounding—acute illness, malnutrition, and statin pre-treatment lowering LDL in the most severe patients—rather than to a protective effect of low LDL itself (Yao et al., 2020), and such mechanisms would not account for the association observed here. Nevertheless, the present LDL–outcome association warrants cautious interpretation. A single LDL measurement obtained within 24 h of admission may not reflect long-term lipid exposure, because acute ischemic stress, inflammation, and reduced nutritional intake can alter circulating lipid levels, so the measured values may partly capture the post-stroke metabolic state rather than pre-morbid atherosclerotic burden. In addition, statin use at admission—which was not systematically recorded in the present dataset—may confound the apparent LDL–outcome association, because statin therapy both lowers LDL and modulates post-stroke inflammatory and recovery cascades (Tan et al., 2024). Future studies should prospectively record statin use, nutritional markers, and inflammatory indices so that the LDL association can be interpreted with greater confidence.

The trajectory of the ipsilesional ALPS index in our cohort provides preliminary, hypothesis-generating evidence that acute perivascular fluid dynamics, as captured by the ipsilesional ALPS index, may contribute prognostic information following IVT. Our univariate results demonstrating a significant association between higher ALPS indices and favorable outcomes corroborate preclinical and clinical reports indicating that ischemic stroke rapidly impairs perivascular clearance, exacerbating cytotoxic edema and neurotoxic waste accumulation (Zeng et al., 2025). Specifically, the reduction in ALPS values observed in acute patients mirrors findings in subcortical infarct populations where glymphatic failure correlates with early cognitive decline, suggesting a shared pathway of impaired interstitial fluid drainage across stroke subtypes (Wang et al., 2025). Mechanistically, this dysfunction is thought to arise from the depolarization of aquaporin-4 channels on astrocytic end-feet, which disrupts the convective flow necessary for clearing metabolic byproducts like beta-amyloid and tau proteins (Mathias et al., 2025).

A question naturally arises from these findings: why did the ipsilesional ALPS index fail to reach conventional significance in the full four-covariate model (*p* = 0.082) yet still yield a significant improvement in nested-model discrimination (ΔAUC = 0.018; likelihood-ratio test *p* = 0.042)? We first note that, for a single added predictor, the Wald test of the regression coefficient and the likelihood-ratio test of the nested model address the same null hypothesis and are asymptotically equivalent; in finite samples, differences between them typically reflect the known conservatism of the Wald test in modest cohort sizes rather than a difference in the hypothesis being tested. Consistent with this, in the three-covariate model used for the nested-model comparison (admission NIHSS score, LDL cholesterol, and the ALPS index), the Wald test of the ALPS coefficient yielded *p* = 0.045, closely matching the likelihood-ratio result (*p* = 0.042), whereas in the full model additionally adjusting for smoking the coefficient was attenuated toward the null (aOR = 1.525, *p* = 0.082). Taken together, these results indicate a small but reproducible prognostic signal. We therefore interpret the incremental value of the ALPS index as statistically detectable but modest, and we emphasize that it should be regarded as hypothesis-generating until confirmed in larger, external cohorts.

The significant negative correlation between the ipsilesional ALPS index and age (*r* = −0.31, *p* < 0.001) in our cohort is consistent with the well-documented age-related decline in glymphatic function reported in both animal models and human neuroimaging studies (Fang et al., 2026). This observation raises an important question: to what extent does the prognostic value of the ALPS index reflect age-related baseline vulnerability versus stroke-induced acute glymphatic dysfunction? Our subgroup analysis addresses this distinction. Although older patients exhibited lower absolute ALPS values, the directional association between higher ALPS indices and favorable outcome was preserved within both age strata (Table 5). Taken together, these findings suggest that stroke-induced acute glymphatic dysfunction—as captured by the ipsilesional ALPS index—represents a pathological process that is at least partially independent of the age-related baseline decline in perivascular clearance. Future studies incorporating age-matched healthy controls would be valuable to more precisely quantify the stroke-attributable component of ALPS reduction and its specific contribution to post-stroke recovery.

Our study has several limitations. Beyond those already noted, several covariates with established prognostic relevance were not systematically collected in the present dataset and therefore could not be entered into the multivariable model; these include occlusion segment (M1 vs. M2), recanalization status after intravenous thrombolysis, DWI infarct volume, hemorrhagic transformation, pre-stroke modified Rankin Scale, diabetes mellitus, atrial fibrillation or stroke etiology, and white matter hyperintensity burden. Among these, infarct volume is likely the most important, because it plausibly influences both functional outcome and perilesional water diffusivity and could therefore confound the apparent ALPS–outcome association. Onset-to-needle time was recorded in this cohort and served as an eligibility criterion (all included patients were treated within 4.5 h of symptom onset) (Figure 2), but it was not entered as a covariate in the present models; it should be incorporated into future prognostic models. We acknowledge these omissions candidly and regard them as a priority for our ongoing and future work, in which these covariates will be prospectively recorded and incorporated into more comprehensive prognostic models. First, our DTI data were acquired under routine clinical protocols and were not corrected for susceptibility-induced distortions, eddy-current artifacts, Gibbs ringing, or thermal noise. While this reflects real-world imaging conditions, it may have introduced measurement variability into the ALPS index. Second, although regions of interest (ROIs) were carefully placed to avoid visible white matter hyperintensities (WMHs), we did not quantitatively assess total WMH burden, which could confound perivascular diffusivity estimates and thus ALPS values. Third, image co-registration between DTI and T2-weighted images relied on a rigid-body (linear) transformation; future studies could explore nonlinear registration to improve anatomical alignment. Beyond methodological considerations, our sample size of 128 patients, while sufficient for preliminary analysis, may have limited statistical power to detect a fully independent effect of the ALPS index—evident from its borderline *p*-value in multivariable regression. Combined with the single-center design, this restricts generalizability to broader stroke populations, such as those with different occlusion sites or receiving endovascular therapy. Furthermore, the prognostic model incorporating ALPS, despite showing statistically significant incremental discrimination (ΔAUC = 0.018, *p* = 0.042) and reclassification improvement (NRI = 0.121, *p* = 0.036), lacks external validation in an independent cohort, precluding robust assessment of its clinical transportability—a critical step for any novel biomarker. Finally, ALPS was measured at a single early time-point, potentially missing dynamic changes in perivascular fluid dynamics during stroke recovery. While the ALPS index correlates with glymphatic activity in preclinical models, it remains an indirect proxy that cannot directly quantify glymphatic flow in humans.

In conclusion, this exploratory prospective cohort suggests that the ipsilesional ALPS index, assessed within 24 h of symptom onset, may serve as a non-invasive MRI marker that provides modest incremental prognostic information beyond conventional clinical indicators (NIHSS and LDL-C) in patients with acute MCA occlusion treated with IVT; the magnitude of this incremental value is small and requires external validation before clinical translation. These findings highlight the potential of glymphatic imaging as a complementary tool for risk stratification. Future research should prioritize external validation in larger, multi-center cohorts and investigate the integration of the ALPS index with other advanced imaging biomarkers, such as collateral status or perfusion parameters, to develop more comprehensive predictive models for personalized stroke management.

## Data Availability

The data analyzed in this study is subject to the following licenses/restrictions: The datasets used and/or analyzed during the current study are available from the corresponding author upon reasonable request, pending approval by the Institutional Review Board of Taixing People’s Hospital and execution of a data use agreement to ensure participant confidentiality. Requests to access these datasets should be directed to Yu Peng, yupeng20150415@163.com.
